# Melatonin Maintains Anabolic-Catabolic Equilibrium and Regulates Circadian Rhythm During Osteoarthritis Development in Animal Models: A Systematic Review and Meta-analysis

**DOI:** 10.3389/fphar.2021.714974

**Published:** 2021-09-17

**Authors:** Dewan Md. Sumsuzzman, Jeonghyun Choi, Zeeshan Ahmad Khan, George Kamenos, Yonggeun Hong

**Affiliations:** ^1^ Department of Rehabilitation Science, Graduate School of Inje University, Gimhae, Korea; ^2^ Biohealth Products Research Center (BPRC), Inje University, Gimhae, Korea; ^3^ Ubiquitous Healthcare and Anti-aging Research Center (u-HARC), Inje University, Gimhae, Korea; ^4^ Department of Physical Therapy, College of Healthcare Medical Science and Engineering, Gimhae, Korea; ^5^ Department of Medicine, Division of Hematology/Oncology, Harvard Medical School and Beth Israel Deaconess Medical Center, Boston, MAUnited States

**Keywords:** melatonin, osteoarthritis, anabolic, catabolic, circadian rhythms, systematic review, meta-analysis

## Abstract

**Background:** The driving force behind osteoarthritis (OA) pathogenesis is an anabolic-catabolic (a/c) imbalance. Melatonin (MT) is a key player in maintaining a/c stability and mitigates OA pathogenesis, but mechanisms underlying its effects remain poorly understood.

**Objectives:** We performed a systematic review analyzing the experimental data that support the clinical applicability of MT in the treatment of OA pathogenesis, placing particular emphasis on the regulation of circadian rhythms and a/c balance.

**Methods:** Major electronic databases and grey literature were used to identify related original articles. Methodological quality of all selected studies was evaluated using the SYRCLE risk of bias tool. Pooled mean differences (MDs)/standardized mean differences (SMDs) with 95% confidence intervals (CIs) were calculated to estimate the effect size.

**Results:** Eleven trials were included in this systematic review. Compared with the control group, MT significantly decreased the levels of interleukin-1β (IL-1β; SMD = −5.45; 95% CI [−6.78, −4.12]; *p* < 0.00001, and histological grading scale (SMD = −3.46; 95% CI, [−5.24, −1.68]; *p* < 0.0001). MT significantly increased the transforming growth factor-β1 (TGF-β1; SMD = 1.17; 95% CI [0.31, 2.03]; *p* < 0.0007). Furthermore, core circadian clock genes *Per2* and *Cry1* mRNA levels were regulated by MT treatment in OA progression.

**Conclusion:** MT may maintain a/c balance and regulate circadian rhythms during OA development. MT could be used in as adjunct with other interventions to manage pain and OA severity.

## Introduction

Osteoarthritis (OA) a commonly diagnosed degenerative joint disease predominantly characterized by progressive degradation of cartilage components, eventually escalating to structural damage and functional failure of the cartilage (Pap and Korb-Pap, 2015; Loeser et al., 2016; Chen et al., 2017). Knee OA (KOA) accounts for about 80% of the burden of OA globally (Global Burden of Disease Study, 2016). In 2020, there were almost 654.1 million individuals (≥40 years) with KOA worldwide (Cui et al., 2020). Aging has always been considered a significant etiological factor for OA (Rahmati et al., 2017), however, the underlying mechanism between age and OA pathogenesis is still not completely understood. Aging contributes to the disruption of anabolic and catabolic cell signaling, leading eventually to osteoarthritic cartilage destruction (Lotz and Loeser, 2012; Loeser, 2017). Understanding the aging process responsible for metabolic dysregulation is critical to identifying and developing effective drugs to treat the pathobiological symptoms of OA. In addition, despite significant efforts over the past several decades to develop KOA therapies, only controversial analgesics (gastrointestinal complications and cardiovascular adverse events) are widely used to treat OA (Pelletier et al., 2016). Hence, targeting a/c balance during aging may provide a novel strategy to prevent/treat cartilage damage in OA.

Imbalance between catabolic and anabolic factors accelerates catabolic activity as a result chondrocytes (major regulators of matrix anabolism-catabolism) completely fail to compensate for the depletion of extracellular matrix (ECM) molecules (Kim et al., 2014; Nummenmaa et al., 2015; Kang et al., 2019). The catabolic phenotype is regulated by several proinflammatory cytokines, including interleukin (IL)-1β, tumor necrosis factor α (TNF-α), and IL-6 (Mueller and Tuan, 2011). IL-1β, a well-known marker for OA progression found in synovial fluid and expressed by both synovial tissue and chondrocytes in osteoarthritic joints (Melchiorri et al., 1998; Mueller and Tuan, 2011). IL-1β triggers the matrix-degrading enzymes such as matrix metalloproteinase (MMP)-13 and suppresses the production of ECM proteins such as collagen type II by chondrocytes (Mobasheri et al., 2017; Wang et al., 2019). Likewise, TNF-α synergistically increases MMPs and reduces ECM proteins (Nambi, 2021). On the other hand, the anabolic phenotype is maintained by a number of growth factors, including transforming growth factor-β1 (TGF-β1), bone morphogenetic protein-2 (BMP-2), and fibroblast growth factors (FGF) (Mueller and Tuan, 2011). TGF-β1 involved in synthesis of collagen type II and downregulates MMPs (Li et al., 2016; Hwang et al., 2020), thereby counteracting IL-1β-mediated cartilage matrix degradation. Interestingly, TGF-β1 gradually declines with aging (van der Kraan, 2017), which may be responsible for cartilage damage in aged cartilage. Recent evidence demonstrated that the misalignment of circadian rhythms is associated with a/c imbalance in various animal models (Gossan et al., 2015; Kc et al., 2015; Hossain et al., 2019). Recent work by Kc et al*.* (Kc et al., 2015) has shown that chronic disruption of circadian rhythms (weekly 12-h phase shift in the light-dark cycle for 22 weeks) induced OA pathology, including upregulation of matrix-degrading enzymes and downregulation of anabolic mediators in the mouse knee joint. Furthermore, the circadian core clock transcription factor *Bmal1* (brain and muscle ARNT-like 1) was downregulated both in human OA and aged mouse cartilage and contributes to disruption in cartilage homeostasis (Dudek et al., 2016). Contrastly, overexpression of *Bmal1* equilibrated the metabolic imbalance of chondrocytes (Yang et al., 2016). Therefore, targeting a/c homeostasis and regulating circadian rhythms by the same candidate drug would be an interesting approach for the treatment of OA.

Melatonin (MT) is a pineal hormone secreted in all vertebrates, including humans, exhibiting anti-oxidant (Sumsuzzman et al., 2020), anti-inflammatory (Ling et al., 2020), and anti-aging (Sumsuzzman et al., 2021a) functions. Endogenous MT levels are inversely proportional to age (Sumsuzzman et al., 2021b) and serum MT declines in OA patients (Oskoii et al., 2020). Additionally, circadian synchronization of MT is dysregulated with advancing age and it closely related to OA etiology (Jahanban-Esfahlan et al., 2018). Several studies showed that MT can protect chondrocyte growth and promote the expression of cartilage-related genes, which may be related to its anti-inflammatory and anti-oxidative stress effects (Pei et al., 2009; Liu et al., 2014). Although MT shows some progress in mitigating OA pathogenesis, many mechanisms underlying its effects remain poorly understood (Hong et al., 2014), and evidence synthesis is needed to justify the specific role of MT in cartilage homeostasis. Furthermore, MT exerts beneficial effects with other interventions, including antiosteoporotic agents, betamethasone, and exercise (Huang et al., 2010b; Hong et al., 2014; Paulino Silva et al., 2021). In OA animal models, however, these combined intervention strategies aimed at a/c homeostasis have not yet been systemically reviewed. Therefore, we conducted a systematic review and meta-analysis aimed at analyzing the experimental data supporting the clinical applicability of MT in the treatment of OA pathogenesis, with particular emphasis on the regulation of circadian rhythms and anabolic-catabolic balance.

## Materials and Methods

### Search Strategy

We performed a comprehensive literature search using major electronic databases, including PubMed, Embase, and China National Knowledge Infrastructure (CNKI) as well as grey literature sources, including Worldcat and Mednar. We identified peer-reviewed studies published up until April 2021 that focused on assessing the impact of exogenous MT in animal models of OA. Furthermore, the reference lists of the included studies and of relevant reviews were examined for additional relevant trials. To obtain animal studies, animal search filters in PubMed and Embase were utilized (Hooijmans et al., 2010; de Vries et al., 2011). The in-depth search strategy performed in PubMed and Embase electronic databases are given in Supplementary Table S1. No limits (e.g., on language or publication date) were used.

### Inclusion Criteria

The inclusion criteria for the selected studies were as follows: 1) the subjects were laboratory animals of any age, sex, or strain used as OA models; 2) the interventions encompassed any type of MT treatment or MT combined with other interventions that was compared with a placebo control, 3) the control intervention consisted of saline, dimethyl sulfoxide, or another vehicle. In addition, the following study outcomes were required for inclusion in the analysis. 1) Primary outcomes: catabolic (i.e., IL-1β, IL-6, TNF-α, MMP-13, ADAMTS-4; a disintegrin and metalloproteinase with thrombospondin motifs-4). and anabolic factors (i.e., TGF-β1, BMP-2, FGF, SOX-9; transcription factor SOX-9, COL2A1; Collagen type II α1) were chosen as the dependent variables to measure the effects of MT in animal models of OA. 2) Secondary outcomes: the effects of MT on circadian regulation and histological scoring in an animal model of OA.

### Exclusion Criteria

The following exclusion criteria were applied for study selection as shown in Supplementary Table S2: 1) all clinical case reports and studies that were solely *In vitro*, 2) not original studies (e.g., editorial or literature review), 3) studies using genetically modified animals, and 4) if used interventions other than MT, studies were excluded.

### Study Selection

After the removal of duplicates, all unique trials were imported into a Rayyan-a web application to allocate the references randomly (Ouzzani et al., 2016). Then, two of the authors individually screened the titles and abstracts to select relevant studies from the randomly allocated references. Finally, the full-texts of the selected articles were evaluated to identify the trials that fulfilled our eligibility criteria. Any disagreement concerning study selection was settled by consultation with the third author. It should be noted that we did not screen for the presence or absence of specific outcome measures during this phase, because often not all outcome measures were described in the abstract.

### Data Extraction

Two authors (DS and ZK) individually extracted the data from each of the included studies. Information related to the authors, publication year, species, age, weight, sample size, animal model, intervention (dose, frequency, and route of administration), and outcome measures were extracted. In studies with multiple arms, only data from the control, MT, MT combined with other intervention groups were considered in this study. If the published outcome data were incomplete, we attempted contacting the authors of the study to obtain the original data. A reminder was sent by email to those who had not responded within 2 weeks. If efforts to achieve the original data failed, the article was eliminated from the meta-analysis. If the data were presented graphically only, GetData Graph Digitizer was employed to extract numerical data from graphs or figures (Sumsuzzman et al., 2021a).

### Assessment of Methodological Quality

The risk of bias (RoB) in the included articles was evaluated by two independent reviewers (DS and JC) using the SYRCLE RoB tool (Hooijmans et al., 2014). Based on the Cochrane RoB tool (Higgins et al., 2011), the RoB tool was developed to evaluate the aspects of bias specifically seen in animal intervention studies. The tool contains 10 items related to six types of bias (selection, performance, detection, attrition, reporting, and other bias). The scores “yes”, “no”, and “unsure” indicate a low, high, and unclear RoB, respectively.

### Data Analysis

The experimental and control group data from the included studies were extracted and inputted into the Review Manager Software (ver. 5.3, The Nordic Cochrane Centre, Copenhagen, Denmark). The meta-analysis was executed when a minimum of two studies were analogous and when these studies provided relevant data (Sumsuzzman et al., 2021a). In the effect size analysis, the mean difference (MD) was used when the outcome measure of all studies employed the same scale, whereas the standardized mean difference (SMD) was used when the studies assessed the same outcome but measured it in different ways (Sumsuzzman et al., 2021a). For both strategies, 95% confidence intervals (CIs) were calculated. The Cochrane Q and I^2^ tests were utilized to assess heterogeneity among the studies; *p* < 0.10 in the Cochrane Q test and I^2^ > 50% were considered to indicate the presence of heterogeneity among the included studies. The fixed-effects model was used for the meta-analysis when statistical heterogeneity had been found lower (I^2^ = < 50%), and the random-effects model was applied when statistical heterogeneity had been found higher (I^2^ = > 50%) (DerSimonian and Laird, 1986). Post-hoc sub-group analysis was performed to investigate the sources of heterogeneity based on wider versus narrow confidence intervals. Subgroup analyses were performed only when subgroups contained at least two independent comparisons. When at least three studies were included, a leave-one-out sensitivity analysis was performed to validate our findings and assess potential sources of heterogeneity (Patsopoulos et al., 2008). Publication bias was analyzed when one outcome variable was associated with at least 10 studies in the meta-analysis, since with <10 studies the power of the tests is too low (Sumsuzzman et al., 2020).

## Results

### Study Search and Selection

**FIGURE 1:**
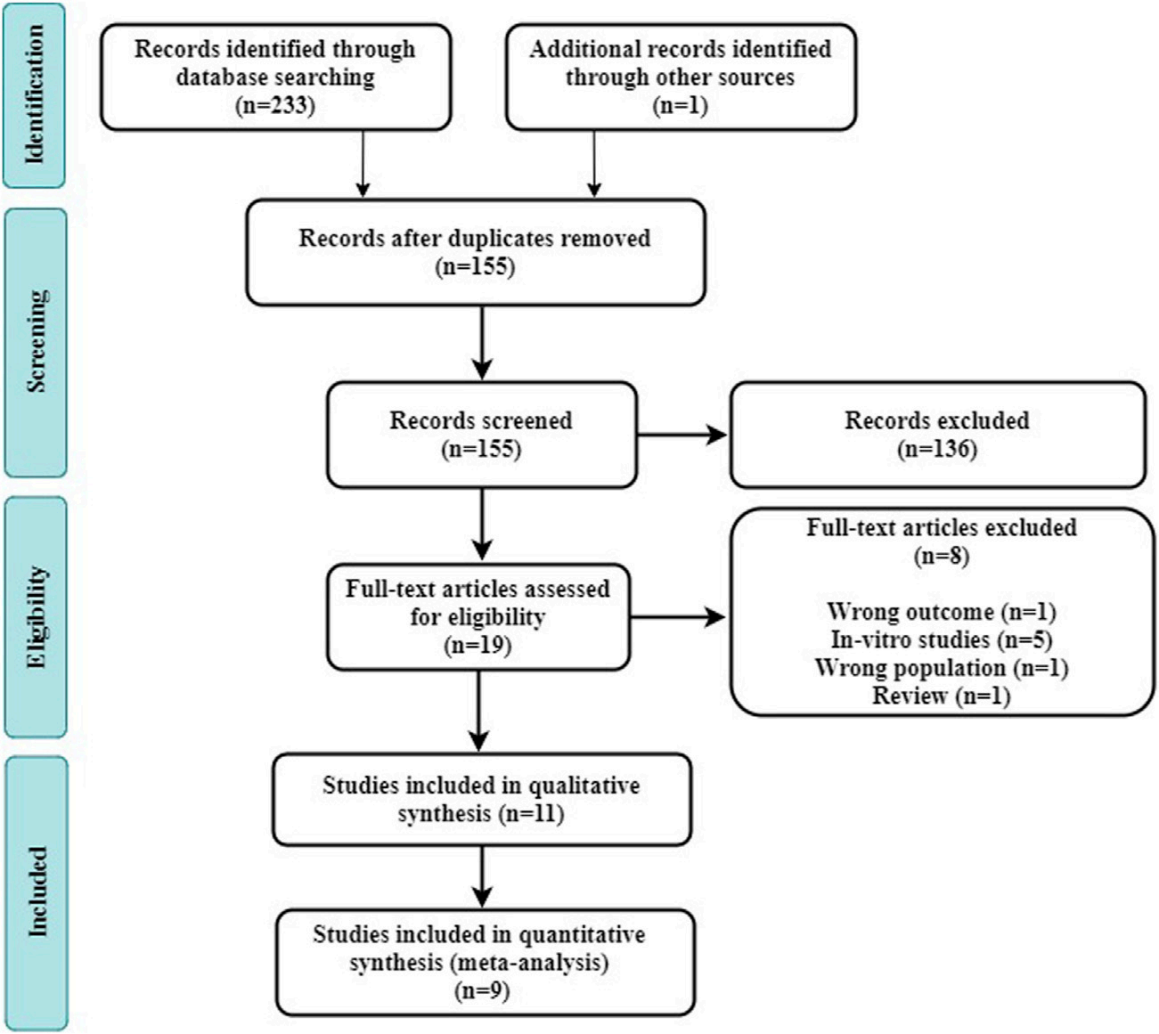
Flow diagram of the systematic review and literature search results of the meta-analysis.

A total of 233 trials (Pubmed = 70, Embase = 122, CNKI = 9, and grey literature databases = 32) were retrieved from the electronic database search. After the removal of duplicates, a total of 155 studies remained, 136 of which were excluded because the titles and abstracts did not meet the eligibility criteria. The remaining 19 studies were selected for full-text analysis, and of these, 11 studies fulfilled our eligibility criteria and were selected for review (Figure 1).

### Study Characteristics

The characteristics of all included studies are reported in Table 1. Three different animal species were used: rats in nine studies (Huang et al., 2010a, 2010b, 2010c; Hong et al., 2014, 2017; Jiang, 2014; Guo et al., 2017; Savtekin et al., 2018; Paulino Silva et al., 2021), mice in one study (Zhang et al., 2019), and rabbits in one study (Lim et al., 2012). In addition, the OA models were created using different methods: collagenase injection in two studies (Hong et al., 2014, 2017), anterior cruciate ligament transection surgery in two (Guo et al., 2017; Paulino Silva et al., 2021), partial medial meniscectomy surgery in one (Lim et al., 2012), intra-articular injection of zymosan in one (Savtekin et al., 2018), destabilization of the medial meniscus in one (Zhang et al., 2019), and injection of papain solution in four studies (Huang et al., 2010a, 2010b, 2010c; Jiang, 2014). MT was administered *via* three different routes: subcutaneously in three studies (Hong et al., 2014, 2017; Paulino Silva et al., 2021), intraperitoneally in one (Guo et al., 2017), and intra-articularly in seven (Huang et al., 2010a, 2010b, 2010c; Lim et al., 2012; Jiang, 2014; Savtekin et al., 2018; Zhang et al., 2019). The data collection period in all studies ranged from 3 to 7 weeks.

**TABLE 1:** Characteristics of the included studies.

Author (Year)	Animal Characteristics			Study Characteristics			Intervention Characteristics		Outcomes
	Species (Sex)	Age (wks)	Weight (gm)	Model	Exp. (n)	Con. (n)	Dose (ROA)	Frequency	
Paulino Silva et al. (2021)	Rat (Male)	9–12	250–320	ACLT	6	6	200 μg/100g (s.c)	Once/daily for 4 weeks	H&E stain
Zhang et al. (2019)	Mice (Male)	9	NR	DMM surgery	10	10	10 mg/mL (i.a)	twice/wk for 4 weeks	Histological grading: OARSI scores
Savtekin et al. (2018)	Rat (Both)	NR	200–250	Zymosan injection	8	8	10 mg/kg (i.a)	Once^a^	Catabolic factor: COX-1, COX-2
Guo et al. (2017)	Rat (Male)	9–10	NR	ACLT	6	6	10 mg/mL (i.p)	3 weeks	Catabolic factor: TNF-α, IL-1β, MMP-13
Hong et al. (2017)	Rat (Male)	8	NR	Collagenase injection	8	8	10 mg/kg (s.c)	twice/daily for 4 weeks	1. Anabolic factor: TGF-β1, SOX-9, and COL2A1
									2. Catabolic factor: TNF-α, MMP-13, VEGF
									3. Circadian gene: *Bmal1, Per2* and *Cry1*
Hong et al. (2014)	Rat (Male)	8	250–285	Collagenase injection	5	5	10 mg/kg (s.c)	twice/daily for 4 weeks	1. Anabolic factor: TGF-β1
									2. Catabolic factor: TNF-α, IL-6, MMP-13, ADAMTS-4
Jiang (2014)	Rat (Male)	4	130–150	Papain solution injection	10	10	20 mg/mL (i.a)	Four/wk for 4 weeks	1. Histological grading: Mankin grading score
									2. Catabolic factor: IL-1β
									3. Anabolic factor: bFGF
									4. Serum melatonin level
Lim et al. (2012)	Rabbit (Female)	24–18	2000–3,000	PMM surgery	5	5	20 mg/kg (i.a)	Once/wk for 4 weeks	1. Histological grading: Mankin grading score
Huang et al. (2010a)	Rat (Male)	4	120–150	Papain solution injection	10	10	20 mg/mL (i.a)	Four/wk for 4 weeks	1. Histological grading: Mankin grading score
									2. Anabolic factor: TGF-β1
									3. Serum melatonin level
Huang et al. (2010b)	Rat (Male)	4	120–150	Papain solution injection	10	10	20 mg/mL (i.a)	Four/wk for 4 weeks	1. Histological grading: Mankin grading score
									2. Anabolic factor: BMP-2
									3. Catabolic factor: IL-1β
Huang et al. (2010c)	Rat (Male)	4	120–150	Papain solution injection	10	10	20 mg/mL (i.a)	Four/wk for 4 weeks	1. Histological grading: Mankin grading score
									2. Serum melatonin level

a intervention has given prior to sacrificing.

Exp, experimental; Con, control; n, sample size; ROA, route of administration; wks, weeks; NR, not reported; ACLT, anterior cruciate ligament transection; DMM, destabilization of the medial meniscus; OARSI, osteoarthritis research society international; i. a, intra-articular; COX-1, cyclooxygenase-1; COX-2, cyclooxygenase-2; TNF-α, tumor necrosis factor-α; IL-1β, interleukin-1β; i. p, intraperitoneal; MMP-13, matrix metalloproteinase-13; IL-6, interleukin-6; s. c, subcutaneous; TGF-β1, transforming growth factor-β1; COL2A1, Collagen type II-alpha1; PMM, partial medial meniscectomy; ADAMTS-4, A disintegrin and metalloproteinase with thrombospondin motifs four; bFGF, basic fibroblast growth factor; BMP-2, bone morphogenetic protein-2.

### Risk of Bias and Quality of Reporting

**FIGURE 2:**
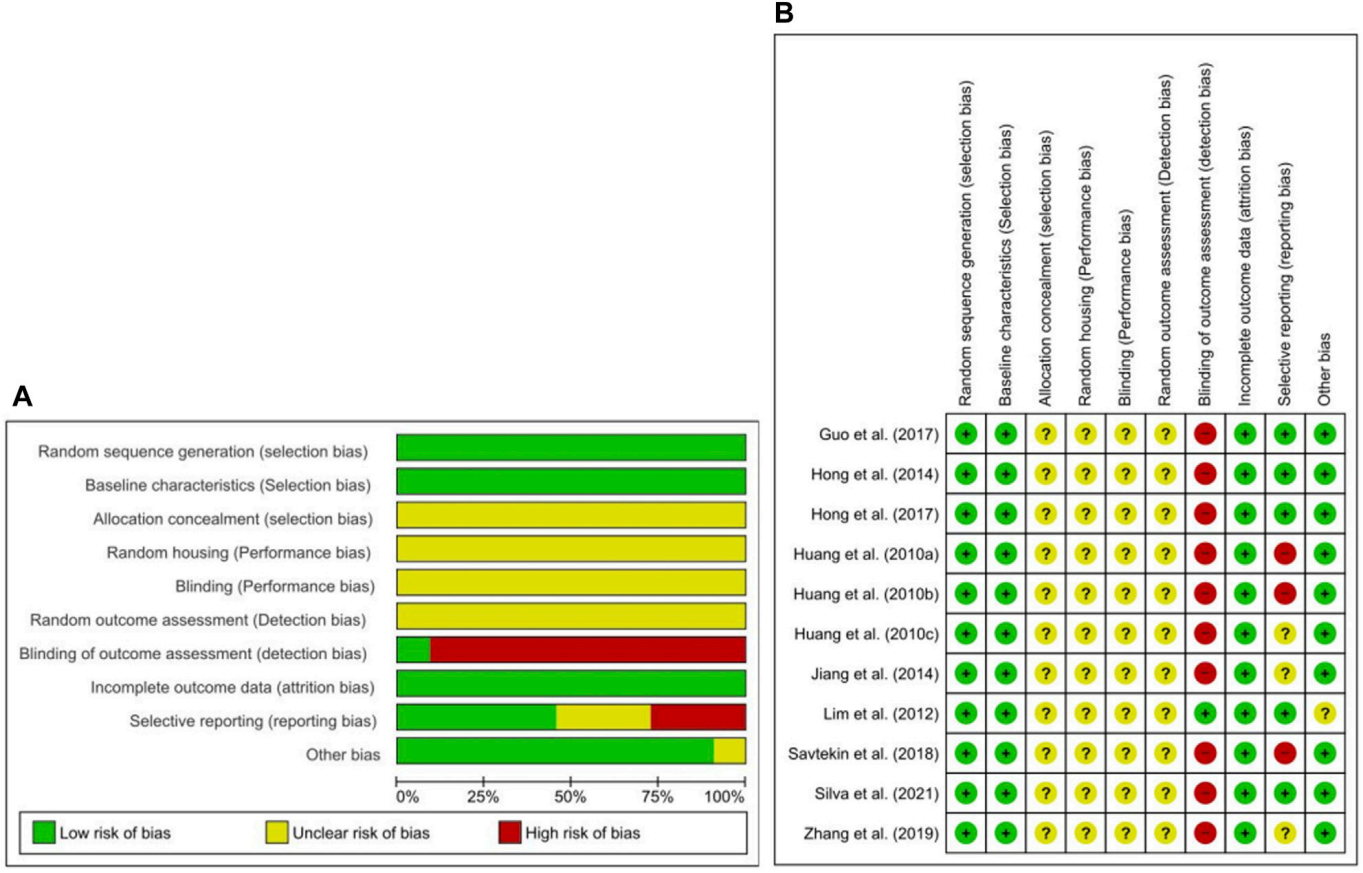
Risk of bias. **(A)** Overall risk of bias for each item in the SYRCLE tool for all included studies. Each risk of bias item is presented as a percentage based on all included studies. **(B)** Individual risk of bias for each of the included animal studies. Each item in the SYRCLE tool was scored as “yes”, “no”, or “unclear”.

The abridged RoB assessment is presented in Figure 2A, and the individual RoB scores of each study are presented in Figure 2B. In all studies, although the animals were randomly allocated, insufficient details regarding the allocation procedure were reported. Baseline characteristics, including sex, age, and weight, were adequately described. Random housing, blinding of caregivers, investigators, and random outcome assessment were partially described in all studies. However, blinding of the outcome assessment showed a high risk of detection bias in nearly all studies. All animals were included in the analyses of all studies. Of the 11 studies, five did not use selective outcome reporting providing sufficient details of the pre-defined outcomes (Lim et al., 2012; Hong et al., 2014, 2017; Guo et al., 2017; Paulino Silva et al., 2021), and remaining six out of three studies had an unclear rating (Huang et al., 2010c; Jiang, 2014; Zhang et al., 2019), and three studies high risk of reporting bias owing to not explicitly reported ethical approval (Huang et al., 2010a, 2010b; Savtekin et al., 2018 ).

### Meta-Analysis

#### Anticatabolic Effect of Melatonin on Osteoarthritis

**FIGURE 3:**
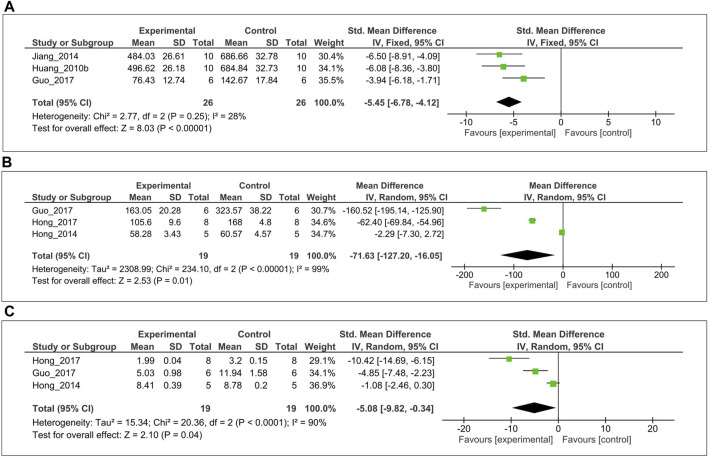
Forest plot showing the effects of MT on IL-1β, TNF-α, and MMP-13 levels. **(A)** IL-1β **(B)** TNF-α, and **(C)** MMP-13 levels. The unit for TNF-α is pg/ml. MT, melatonin; IL-1β, interleukin-1β; TNF-α, tumor necrosis factor-α; MMP-13, matrix metalloproteinase-13; CI, confidence interval.

The inhibitory effect of MT on the level of IL-1β over a 3–4-week period was evaluated in three studies (Huang et al., 2010c; Jiang, 2014; Guo et al., 2017). Of these, left cartilage of femoral condyle IL-1β level was measured from two studies (Huang et al., 2010c; Jiang, 2014), one study (Guo et al., 2017) from synovial fluid. For the meta-analysis, biochemical outcomes were pooled and analyzed using a fixed-effect model **(** Figure 3A **)**. The analysis showed that MT significantly reduced the IL-1β level compared with the control (SMD = −5.45; 95% CI [−6.78, −4.12]; I^2^ = 28%, *p* < 0.00001). Similarly, anticatabolic effects of MT on the level of TNF-α over a 3–4-week period was evaluated in three studies (Hong et al., 2014, 2017; Guo et al., 2017). Of these, serum TNF-α level was measured from two studies (Hong et al., 2014, 2017), one study (Guo et al., 2017) from synovial fluid. For the meta-analysis, biochemical outcomes were pooled and analyzed using a random-effects model **(** Figure 3B **)**. The analysis showed that MT significantly reduced the TNF-α level compared with the control (MD = −71.63; 95% CI [−127.20, −16.05]; I^2^ = 99%, *p* = 0.01). Finally, the inhibitory effect of MT on the MMP-13 level in articular cartilage was evaluated in three studies (Hong et al., 2014, 2017; Guo et al., 2017). For the meta-analysis, biochemical outcomes were pooled and analyzed using a random-effect model **(** Figure 3C **)**. The analysis showed that MT significantly reduced the MMP-13 level compared with the control (SMD = −5.08; 95% CI [−9.82 to −0.34]; I^2^ = 90%, *p* = 0.04).

#### Pro-anabolic Effect of Melatonin on Osteoarthritis

**FIGURE 4:**
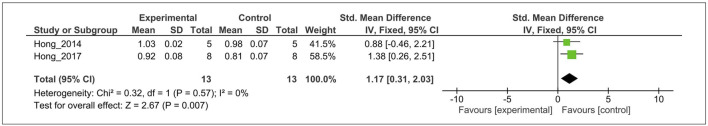
Forest plot showing the effects of MT on TGF-β1 levels. MT, melatonin; TGF-β1, transforming growth factor-β1; CI, confidence interval; IV, independent variable.

The anabolic effect of MT on the level of TGF-β1 was evaluated in cartilage tissue from two studies (Hong et al., 2014, 2017). For the meta-analysis, biochemical outcomes were pooled and analyzed using a fixed-effect model **(** Figure 4 **)**. The analysis showed that MT significantly increased the TGF-β1 expression compared with the control (SMD = 1.17; 95% CI [0.31, 2.03]; I^2^ = 0%, *p* < 0.0007).

#### Effect of Melatonin on Histological Grading Score

**FIGURE 5:**
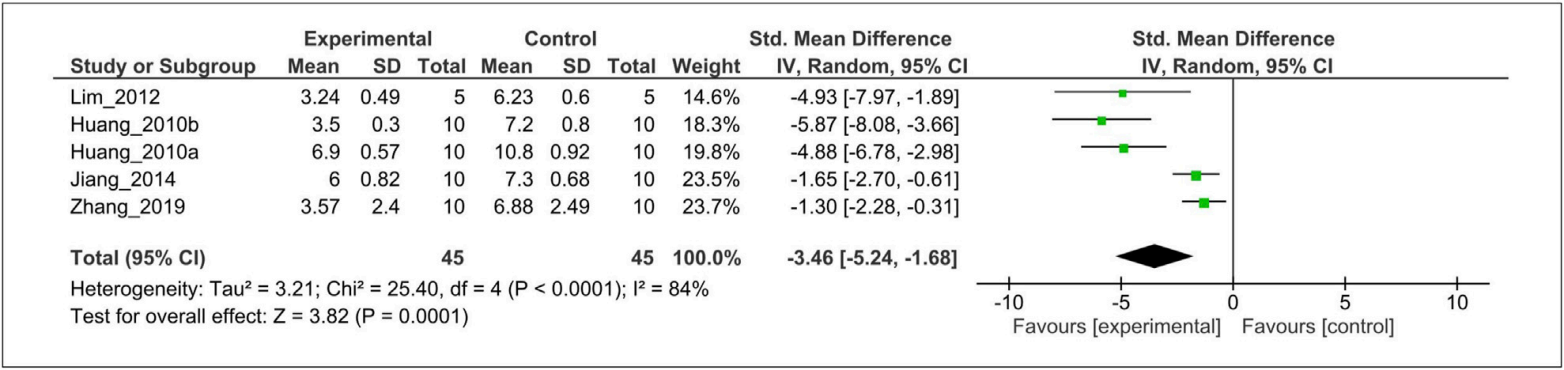
Forest plot showing that MT treatment improved the histological score in OA models. Mankin grading score was used to assess the histological score almost all studies, except Zhang *et al.* (OARSI scores). OA, osteoarthritis; OARSI, osteoarthritis research society international; I, heterogeneity; CI, confidence interval; SD, standard deviation; IV, independent variable.

The effect of MT on the histological score was evaluated in five studies (Huang et al., 2010a, 2010c; Lim et al., 2012; Jiang, 2014; Zhang et al., 2019). For the meta-analysis, histological outcomes were pooled and analyzed using a random-effects model **(** Figure 5 **)**. The analysis showed that MT significantly reduced the histological scoring compared with the control (SMD = −3.46; 95% CI [−5.24, −1.68]; I^2^ = 84%, *p* < 0.0001).

#### Serum Melatonin Levels in Osteoarthritis

**FIGURE 6:**
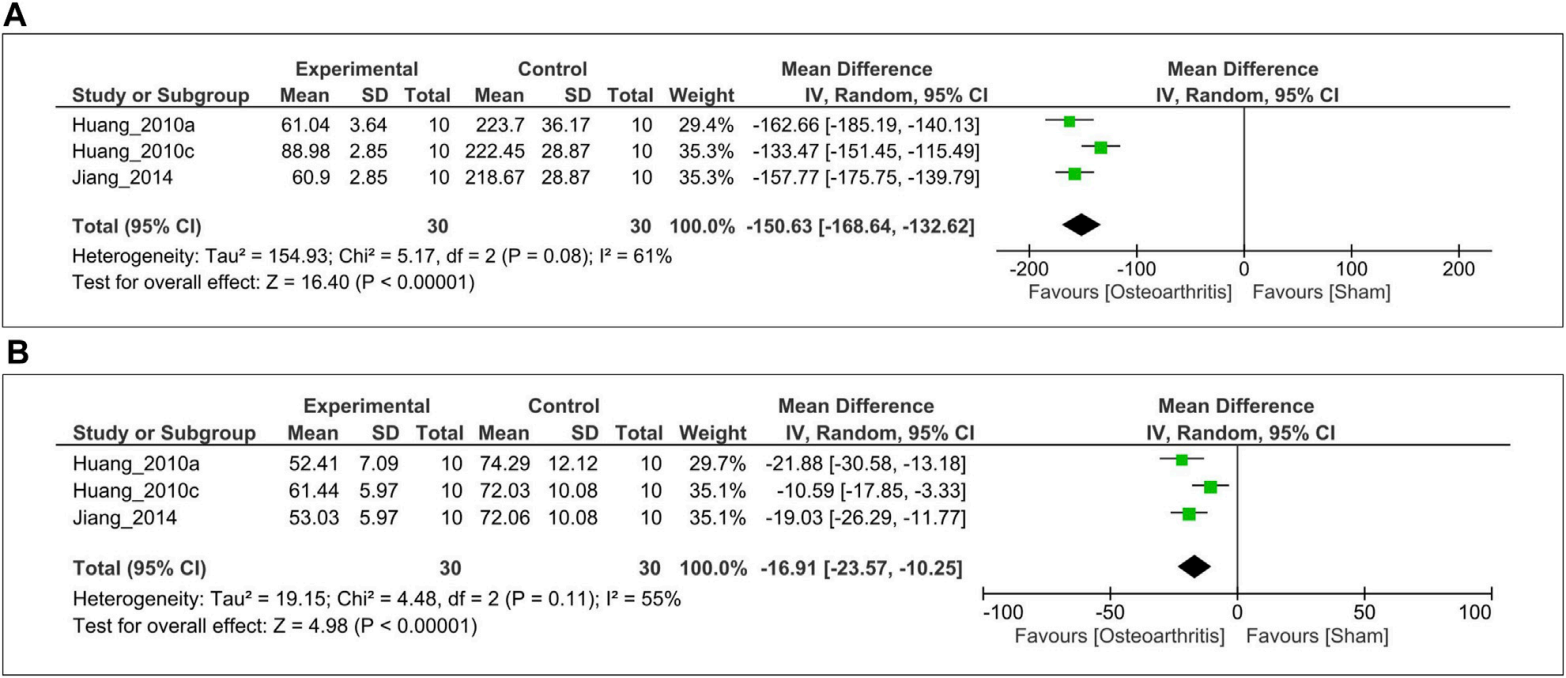
Forest plot showing that serum MT levels. **(A)** 2 AM, and **(B)** 2 PM. The unit for MT is pg/ml. MT, melatonin; CI, confidence interval, IV, independent variable.

The serum MT levels in OA and sham control groups were measured at two time points (night = 2AM, day = 2PM) in three studies (Huang et al., 2010a, 2010b; Jiang, 2014). For the meta-analysis, serum MT level was pooled and analyzed using a random-effects model. The analysis showed that serum MT level significantly reduced in OA animals compared with the sham control at mid-night (SMD = −150.63; 95% CI [−186.64, −132.62,]; I^2^ = 61%, *p* < 0.00001, Figure 6A). Likewise, serum MT concentration declined in OA animals at day time (SMD = −16.91; 95% CI [−23.57, −10.25]; I^2^ = 55%, *p* < 0.00001, Figure 6B).

### Subgroup Analysis

**FIGURE 7:**
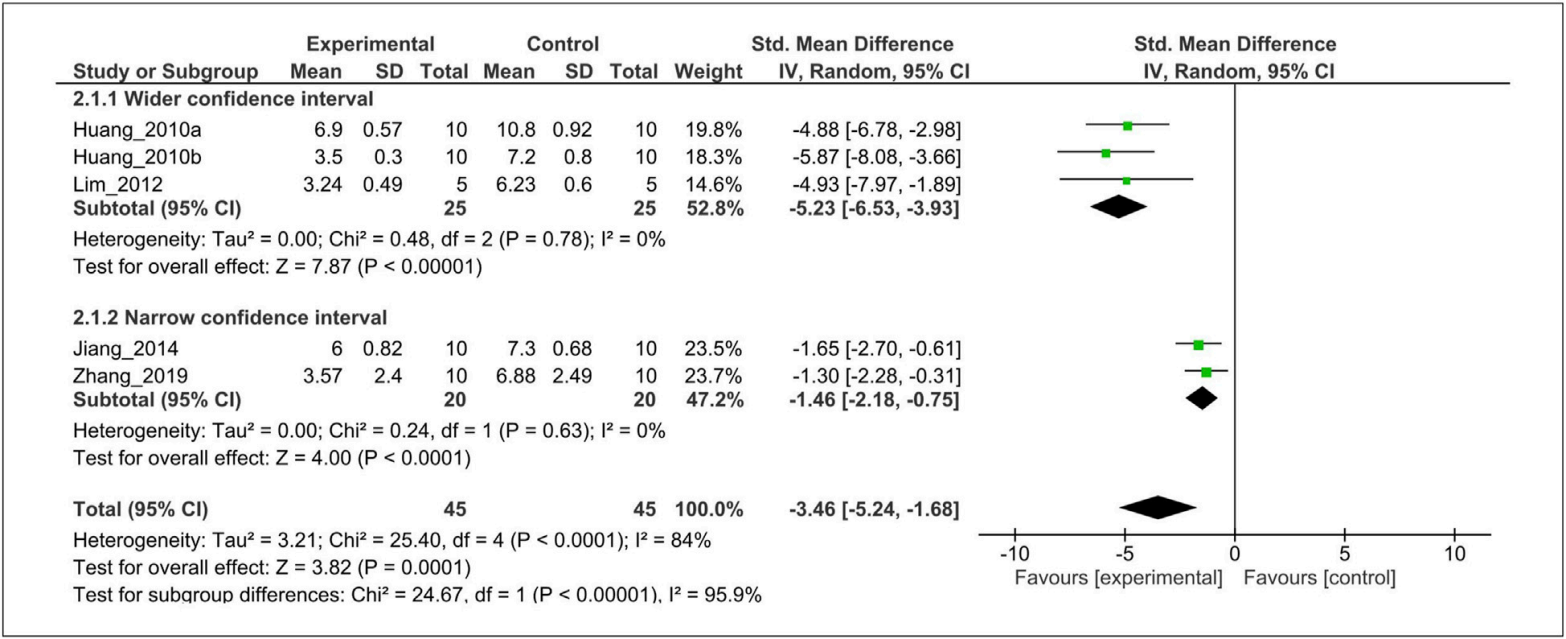
Subgroup analysis of histological score stratified according to the wide 95% CI *versus* narrow 95% CI. The test for subgroup differences showed a statistically significant subgroup effect (*p* < 0.00001), indicating that the wide 95% CI subgroup produced larger effects. Furthermore, there is no heterogeneity between the trials within each of these subgroups (wider-CI: I^2^ = 0%; narrow-CI: I^2^ = 0%). CI, confidence interval; SD, standard deviation; IV, independent variable.

Subgroup analysis was performed to investigate heterogeneity among the studies. With regard to histological grading score, we found considerable heterogeneity (I^2^ = 84%). Thus, we performed post-hoc subgroup analysis based on wider-CI versus narrow-CI. The test for subgroup differences indicated a statistically significant subgroup effect (*p* < 0.00001), implying that wider-CI subgroup relatively produced a larger effect size than the narrow-CI subgroup. Importantly, there is no heterogeneity between the trials within each of these subgroups (wider-CI: I^2^ = 0%; narrow-CI: I^2^ = 0%) (Figure 7).

### Sensitivity Analysis

Sensitivity analysis by excluding individual studies revealed that the IL-1β (Supplementary Table S3), and TNF-α (Supplementary Table S4), MMP-13 (Supplementary Table S5), and histological grading score (Supplementary Table S6) results were not modified when compared to the overall effect and their heterogeneity, indicating that our results were robust and that similar results could be obtained after excluding any of the included studies. The sensitivity analysis based on serum MT level both time points indicated that heterogeneity decreased (I^2^ = 0%) when reference (Huang et al., 2010b) was excluded; the adjusted effect size were MD = −159.67; 95% CI [−173.73, −145.62] and MD = −20.20; 95% CI [−25.78, −14.62] at 2 AM and 2 PM, respectively (Supplementary Table S7, S8).

### Systematic Review

#### Effects of Melatonin on Others Anabolic-Catabolic Factors

Inflammatory cytokines, including IL-6, appear at higher concentrations in the OA joint and are the major contributors of altered chondrocyte function and cartilage degeneration (Hong et al., 2014). Our group has previously shown that serum IL-6 concentration was higher in collagenase-induced OA rats compared with control, and MT treatment significantly attenuated IL-6 level (*p* < 0.05) (Hong et al., 2014). Increased vascular endothelial growth factor (VEGF) levels are involved in OA-specific pathologies such as cartilage damage, osteophyte formation, and pain (Hamilton et al., 2016). Recently, Hong *et al.*(Hong et al., 2017) demonstrated that treatment with MT notably decreased VEGF level in an OA animal model when compared with a vehicle-treated group (*p* < 0.05). Aggrecanase-1 (ADAMTS-4) inhibitors are considered to have potential therapeutic strategy in the treatment of OA as their expression is elevated during OA progression and they are responsible for cartilage damage (Verma et al., 2016). Our colleagues also previously disclosed that ADAMTS-4 level 1.5 times higher in the collagenase-induced OA group than the control group, whereas MT treatment markedly attenuated ADAMTS-4 (*p* < 0.05) (Hong et al., 2014). In the past, it has been shown that the COX-2 enzyme plays a significant role in the development of OA pathogenesis. Savtekin *et al.* (Savtekin et al., 2018) corroborated with this finding and reported that MT treatment blocked the COX-2 enzymatic activity in the zymosan-induced OA animal model. However, MT treatment was not showed statistically significant in reducing of COX-1 level (Savtekin et al., 2018).

In OA, mRNA expression of many anabolic factors such as SOX-9, and COL2A1 gradually decreased, leading to cartilage damage (Zhong et al., 2016). Interestingly, Hong *et al.* (Hong et al., 2017) reported that MT treatment suppressed SOX-9, and COL2A1 mRNA expression in collagenase-induced OA cartilage compared with control. Conflicting evidence from the same group of investigators show that MT may exert it’s protective effects against OA by increasing COL2A1 protein synthesis (Hong et al., 2014). Upregulation of anabolic factors, including BMP-2 and basic fibroblast growth factor (bFGF) are also associated with cartilage repair in the pathological features of OA (Li et al., 2013; Chang et al., 2015) and previous studies reported that MT treatment significantly elevated BMP-2 and bFGF in OA rats (Huang et al., 2010c; Jiang, 2014).

#### Melatonin Combined With Other Interventions

The anti-arthritic effects of MT combined with strontium ranelate, 5-Methoxytryptophol, exercise, and betamethasone interventions were evaluated on the articular cartilage of OA in rats. Savtekin *et al.* (Savtekin et al., 2018) reported that MT combined with 5-Methoxytryptophol notably downregulated COX-2 when compared with MT alone (*p* < 0.001). Although MT in combination with exercise reduced the level of catabolic enzymes such as MMP-13, catabolic cytokines including TNF-alpha and IL-6 were present significantly higher levels (Hong et al., 2014). Besides, there was a significant difference in histological grading score between MT plus betamethasone and control groups (*p* < 0.05) (Huang et al., 2010b). Silva *et al.* (Paulino Silva et al., 2021) reported administration of the MT combined with strontium ranelate presented chondroprotective effects.

#### Modulatory Effects of Melatonin in Circadian Rhythms

The relationship between circadian misalignment and increase in catabolic activities commonly appear during aging, which may contribute to increasing OA susceptibility (Gossan et al., 2015). Hong et al. reported that several catabolic factors, including MMP-13, and VEGF were upregulated in OA cartilage when the core circadian clock components such as *Per2* (period circadian regulator 2) and *Cry1* (Cry1 cryptochrome circadian regulator 1) genes were decreased (Hong et al., 2017). In contrast, MT treatment not only reduced the catabolic factors in cartilage tissue but also upregulated *Per2* and mRNA levels than the vehicle-treated group (Hong et al., 2017). However, the expression of *Bmal1* was unchanged with the MT-treated group (Hong et al., 2017).

## Discussion

Preclinical trials are typically mandatory for developing and evaluating an intervention prior to performing clinical trials in humans. In addition, systematic reviews are crucial for translating the results from preclinical to clinical trials (Lemon and Dunnett, 2005). The principal aim of this work was to provide evidence for the therapeutic potential of MT in OA pathogenesis by the maintenance of a/c homeostasis and modulation of circadian rhythms. To our best knowledge, this is the first systematic review and meta-analysis concerning the effects of MT on a/c balance and modulation of circadian rhythms in OA. Our results suggest that MT treatment increases TGF-β1 (anabolic factor), reduces catabolic factors (IL-1β, TNF-α, and MMP-13), and reduces histological grading score in OA animals. Furthermore, serum MT level significantly declined in osteoarthritic animals than the sham control group both at midnight and middle of the day. Additionally, others anabolic factors (SOX-9, COL2A1, BMP-2, and bFGF) and catabolic factors (IL-6, VEGF, ADAMTS-4, and COX-2), combined effects of MT, and modulatory effects of MT in circadian rhythms were systematically reviewed in the OA animal models.

Accumulating evidence indicates that several proinflammatory cytokines, including IL-1β, TNF-α, and IL-6, trigger catabolic OA development (Mueller and Tuan, 2011). Particularly, IL-1β plays a critical role in OA and is responsible for the further induction of various catabolic enzymes, and inflammatory mediators (Jia et al., 2017). Studies showed that elevated IL-1β was observed in both synovial fluid and cartilage of OA patients (Melchiorri et al., 1998). In this study, our results showed that MT significantly inhibited the level of IL-1β. In addition, MMP-13, and TNF-α production were also inhibited by MT treatment. The results suggested that MT may protect OA by inhibiting proinflammatory cytokines and catabolic enzymes. These results are consistent with the findings of Guo et al. (2017), who found that MT treatment could inhibit IL-1β, TNFα and suppress MMP-13 in OA (Guo et al., 2017). However, data related to TNF-α must be interpreted cautiously because there was substantial heterogeneity (I^2^ = 99%) and very wide (95%) CI. One possible explanation for these high degrees of heterogeneity and wider 95% CI is that MT may exert consistently better protective effects in OA pathogenesis where pathogenesis is strongly associated with circadian disruption. For example, Guo *et al.* (Guo et al., 2015) has shown that exposure to IL-1β severely altered circadian clock gene expression in cartilage, but TNF-α did not have this type of association. Furthermore, others catabolic factors such as IL-6, VEGF, and ADAMTS-4 have been identified as critical players in the pathogenesis and progression of OA (Mueller and Tuan, 2011; Hamilton et al., 2016; Verma et al., 2016), which are also attenuated by MT treatment (Hong et al., 2014, 2017). Our systematic review has reported that MT treatment suppressed COX-2 enzymatic activity in an OA animal model, although the COX-1 level remains unchanged (Savtekin et al., 2018). Previous work demonstrates that COX-2, but not COX-1 induced by catabolic mediators and COX-2 inhibitor showed the most beneficial effects against articular tissue inflammation, whereas COX-1 inhibitor produced unwanted gastrointestinal side effects (Hawkey, 1999; Martel-Pelletier et al., 2003). Hence, MT can be the drug of choice to manage OA pain due to most nonsteroidal anti-inflammatory drugs (NSAIDs) have well-known adverse effects (affecting the gastric mucosa, renal system, cardiovascular system, hepatic system, and hematologic system (Ghlichloo and Gerriets, 2021)).

In the present study, we found that MT might have a protective effect against OA pathogenesis through the upregulation of TGF-β1. Likewise, BMP-2, and bFGF are also unregulated by MT treatment and shown beneficial effects in OA. These findings may be associated with significant reduction of several catabolic factors and regulation of circadian rhythm pathway. Recent studies also support our idea that elevated expression of catabolic cytokines and disruption of circadian core clock genes are responsible for altered anabolic signaling that eventually promotes OA pathogenesis (Akagi et al., 2017; Hong et al., 2017; Tang et al., 2017; Fu et al., 2019; Hu et al., 2020). In addition, our meta-analysis demonstrated that the serum MT level was notably decreased in OA animals both mid-night (2AM) and middle of the day (2PM). The results further strengthen our hypothesis that targeting a/c homeostasis and regulating circadian rhythms by MT would be a promising approach for the treatment of OA. Even though Hong *et al.* (Hong et al., 2017) reported that *Per2* and *Cry1* were upregulated by MT treatment in OA cartilage, the *Bmal1* expression pattern remains unchanged. Interestingly, Fu et al. (2019) (Fu et al., 2019) demonstrated that exogenous MT upregulated *Bmal1*, but downregulated *Per1*, in chondrocytes. Further studies are required to investigate the differential role of MT in different stages of OA pathogenesis. In our systematic review, we reported MT treatment reduced SOX-9 and COL2A1 mRNA expression in one study (Hong et al., 2017), but another study disclosed that COL2A1 protein level increased by MT (Hong et al., 2014). A further study with more focus on mRNA and protein levels of several anabolic factors, including SOX-9 and COL2A1 is therefore suggested.

Another important finding was that MT significantly reduced the histological score when compared to the control group (*p* < 0.0001), but there was statistically significant heterogeneity (I^2^ = 84%). Thus, we performed subgroup analysis according to wider-CI versus narrow-CI. Our subgroup analysis indicates that the wider-CI subgroup produced relatively a larger effect size than the narrow-CI subgroup. Importantly, there is no heterogeneity between these subgroups (I^2^ = 0%). This finding suggests that MT can be an attractive candidate for the treatment of OA pathogenesis.

In this systematic review, we made great efforts to obtain relatively objective results. First, we searched two legitimate major electronic databases along with Chinese and grey literature databases, and two authors independently examined the retrieved trials to ensure that all relevant studies were incorporated in the meta-analysis. Secondly, two authors assessed the methodological quality of the studies to decrease bias of the included trials and eventually extracted the relevant data. Furthermore, the data extraction was executed by two different authors to ascertain that all of the data outputs were accurately extracted and synthesized for the meta-analysis.

However, this review had several limitations. Although the potential source of heterogeneity has been investigated through subgroup analysis, and leave-one-out sensitivity analyses, there was substantial unexplained heterogeneity between trials for TNF-α and MMP-13 outcomes. Further work is required to know whether gender and age variables modify the MT effects for TNF-α and MMP-13. Our risk of bias analysis using the SYRCLE RoB tool indicated that all included studies had a high risk of detection bias. Information regarding key measures essential for bias reduction (e.g., allocation concealment, random outcome assessment, and blinding) was often missing or insufficiently reported. We strongly recommend improvements to the reporting system for animal models to reduce the risk of bias. Also, deficiency of hormones such as estrogen are directly associated the OA (Roman-Blas et al., 2009). These hormones are also associated with the level of melatonin, catabolic, and anabolic factors. Thus, a research is warranted to explore the role of estrogen and associated hormones, with and without melatonin.

**FIGURE 8:**
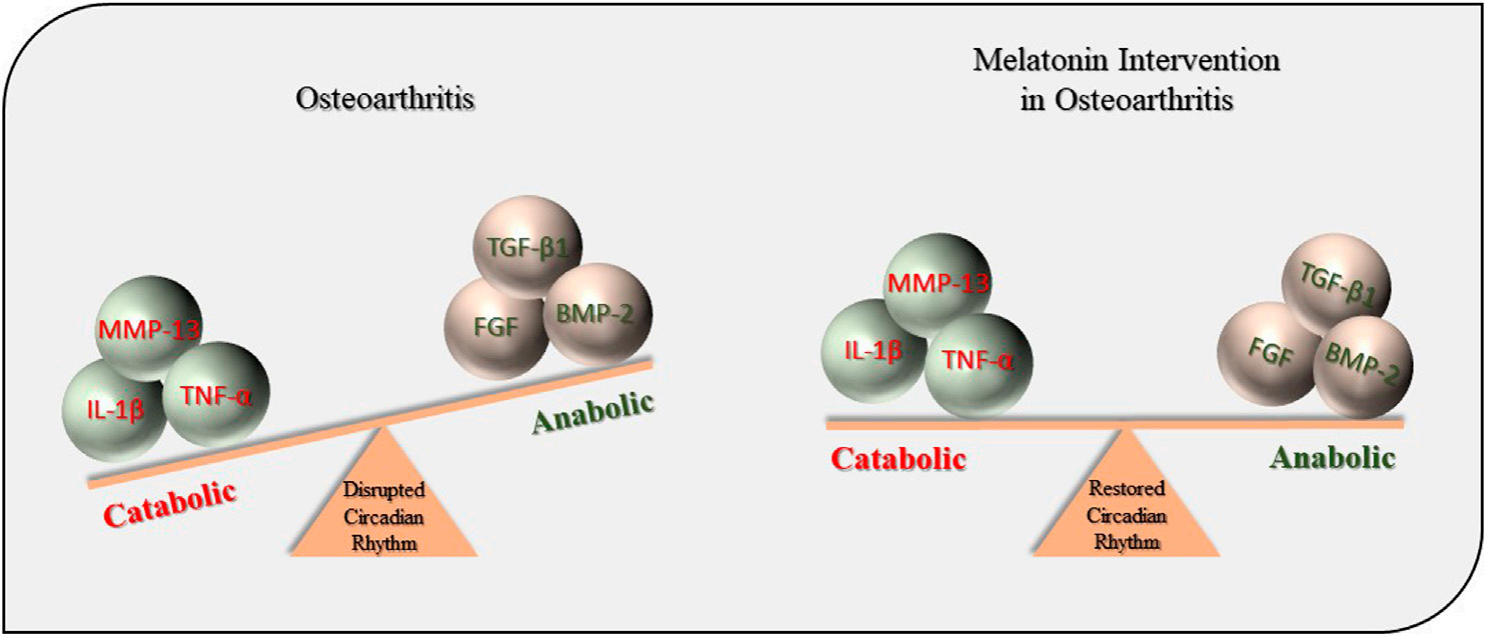
Protective effects of MT during OA pathogenesis. MT, melatonin; OA, osteoarthritis; IL-1β, interleukin-1β; TNF-α, tumor necrosis factor-α; MMP-13, matrix metalloproteinase-13; TGF-β1, transforming growth factor-β1; FGF, fibroblast growth factors; BMP-2, bone morphogenetic protein-2.

In summary, this current study demonstrates that exogenous MT prevents OA pathogenesis through the regulation of circadian rhythms and a/c balance (Figure 8). However, these results should be interpreted in light of the limitations in methodological quality of the studies included in the meta-analysis. Therefore, further studies are warranted to improve study quality through better experimental design and reducing the bias in animal trials.

## Data Availability

The original contributions presented in the study are included in the article/Supplementary Material, further inquiries can be directed to the corresponding author, upon reasonable request.
