# Supplementary material for: Melatonin Maintains Anabolic-Catabolic Equilibrium and Regulates Circadian Rhythm During Osteoarthritis Development in Animal Models: A Systematic Review and Meta-analysis
Source: Front Pharmacol. 2021 Sep 17;12:714974. doi: 10.3389/fphar.2021.714974 (PMC8484877; doi:10.3389/fphar.2021.714974)
Supplement: Supplementary file 1 [file DataSheet1.doc]

# **Supplementary Material**

## **Supplementary Table S1.** Search strategy conducted in PubMed up to April 2021.

### Search strategy for PubMed

| **Search** | **Query** | **Items found** |
| --- | --- | --- |
| #1 | "melatonin"[MeSH Terms] OR "receptors, melatonin"[MeSH Terms] OR "tasimelteon"[Supplementary Concept] OR "ramelteon"[Supplementary Concept] OR "S-20098"[Supplementary Concept] OR "melatonin*"[Text Word] OR "tasimelteon"[Text Word] OR "ramelteon"[Text Word] OR "S-20098"[Text Word] OR "S20098"[Text Word] OR "circadin"[Text Word] OR "rozerem"[Text Word] OR "hetlioz"[Text Word] OR "valdoxan"[Text Word] OR "thymanax"[Text Word] OR "Melovine"[Text Word] OR "5-methoxy-n-acetyltryptamine"[Text Word] OR "n-acetyl-5-methoxytryptamine"[Text Word] OR "MT"[Title/Abstract] OR "MLT"[Title/Abstract] | 67,081 |
| #2 | "osteoarthritis"[MeSH Terms] OR "osteoarthriti*"[Title/Abstract] OR "osteo arthriti*"[Title/Abstract] OR "osteoarthrotic"[Title/Abstract] OR "osteoarthros*"[Title/Abstract] OR "coxarthrosis"[Title/Abstract] OR "OA"[Title/Abstract] | 110,431 |
| #3 | "animal experimentation"[MeSH Terms] OR "models, animal"[MeSH Terms] OR "invertebrates"[MeSH Terms] OR "Animals"[MeSH Terms:noexp] OR "animal population groups"[MeSH Terms] OR "chordata"[MeSH Terms:noexp] OR "chordata, nonvertebrate"[MeSH Terms] OR "vertebrates"[MeSH Terms:noexp] OR "amphibians"[MeSH Terms] OR "birds"[MeSH Terms] OR "fishes"[MeSH Terms] OR "reptiles"[MeSH Terms] OR "mammals"[MeSH Terms:noexp] OR "primates"[MeSH Terms:noexp] OR "artiodactyla"[MeSH Terms] OR "carnivora"[MeSH Terms] OR "cetacea"[MeSH Terms] OR "chiroptera"[MeSH Terms] OR "elephants"[MeSH Terms] OR "hyraxes"[MeSH Terms] OR "insectivora"[MeSH Terms] OR "lagomorpha"[MeSH Terms] OR "marsupialia"[MeSH Terms] OR "monotremata"[MeSH Terms] OR "perissodactyla"[MeSH Terms] OR "rodentia"[MeSH Terms] OR "scandentia"[MeSH Terms] OR "sirenia"[MeSH Terms] OR "haplorhini"[MeSH Terms:noexp] OR "strepsirhini"[MeSH Terms] OR "platyrrhini"[MeSH Terms] OR "tarsii"[MeSH Terms] OR "catarrhini"[MeSH Terms:noexp] OR "cercopithecidae"[MeSH Terms] OR "hylobatidae"[MeSH Terms] OR "hominidae"[MeSH Terms:noexp] OR "gorilla gorilla"[MeSH Terms] OR "pan paniscus"[MeSH Terms] OR "pan troglodytes"[MeSH Terms] OR "pongo pygmaeus"[MeSH Terms] OR (("Animals"[Title/Abstract] OR "animal"[Title/Abstract] OR "mice"[Title/Abstract] OR "mus"[Title/Abstract] OR "mouse"[Title/Abstract] OR "murine"[Title/Abstract] OR "woodmouse"[Title/Abstract] OR "rats"[Title/Abstract] OR "rat"[Title/Abstract] OR "murinae"[Title/Abstract] OR "muridae"[Title/Abstract] OR "cottonrat"[Title/Abstract] OR "cottonrats"[Title/Abstract] OR "hamster"[Title/Abstract] OR "hamsters"[Title/Abstract] OR "cricetinae"[Title/Abstract] OR "rodentia"[Title/Abstract] OR "rodent"[Title/Abstract] OR "rodents"[Title/Abstract] OR "pigs"[Title/Abstract] OR "pig"[Title/Abstract] OR "swine"[Title/Abstract] OR "swines"[Title/Abstract] OR "piglets"[Title/Abstract] OR "piglet"[Title/Abstract] OR "boar"[Title/Abstract] OR "boars"[Title/Abstract] OR "sus scrofa"[Title/Abstract] OR "ferrets"[Title/Abstract] OR "ferret"[Title/Abstract] OR "polecat"[Title/Abstract] OR "polecats"[Title/Abstract] OR "mustela putorius"[Title/Abstract] OR "guinea pigs"[Title/Abstract] OR "guinea pig"[Title/Abstract] OR "cavia"[Title/Abstract] OR "callithrix"[Title/Abstract] OR "marmoset"[Title/Abstract] OR "marmosets"[Title/Abstract] OR "cebuella"[Title/Abstract] OR "hapale"[Title/Abstract] OR "octodon"[Title/Abstract] OR "chinchilla"[Title/Abstract] OR "chinchillas"[Title/Abstract] OR "gerbillinae"[Title/Abstract] OR "gerbil"[Title/Abstract] OR "gerbils"[Title/Abstract] OR "jird"[Title/Abstract] OR "jirds"[Title/Abstract] OR "merione"[Title/Abstract] OR "meriones"[Title/Abstract] OR "rabbits"[Title/Abstract] OR "rabbit"[Title/Abstract] OR "hares"[Title/Abstract] OR "hare"[Title/Abstract] OR "diptera"[Title/Abstract] OR "flies"[Title/Abstract] OR "fly"[Title/Abstract] OR "dipteral"[Title/Abstract] OR "drosophila"[Title/Abstract] OR "drosophilidae"[Title/Abstract] OR "cats"[Title/Abstract] OR "cat"[Title/Abstract] OR "carus"[Title/Abstract] OR "felis"[Title/Abstract] OR "nematoda"[Title/Abstract] OR "nematode"[Title/Abstract] OR "nematodes"[Title/Abstract] OR "sipunculida"[Title/Abstract] OR "dogs"[Title/Abstract] OR "dog"[Title/Abstract] OR "canine"[Title/Abstract] OR "canines"[Title/Abstract] OR "canis"[Title/Abstract] OR "sheep"[Title/Abstract] OR "sheeps"[Title/Abstract] OR "mouflon"[Title/Abstract] OR "mouflons"[Title/Abstract] OR "ovis"[Title/Abstract] OR "goats"[Title/Abstract] OR "goat"[Title/Abstract] OR "capra"[Title/Abstract] OR "capras"[Title/Abstract] OR "rupicapra"[Title/Abstract] OR "chamois"[Title/Abstract] OR "haplorhini"[Title/Abstract] OR "monkey"[Title/Abstract] OR "monkeys"[Title/Abstract] OR "anthropoidea"[Title/Abstract] OR "anthropoids"[Title/Abstract] OR "saguinus"[Title/Abstract] OR "tamarin"[Title/Abstract] OR "tamarins"[Title/Abstract] OR "leontopithecus"[Title/Abstract] OR "hominidae"[Title/Abstract] OR "ape"[Title/Abstract] OR "apes"[Title/Abstract] OR "pan paniscus"[Title/Abstract] OR "bonobo"[Title/Abstract] OR "bonobos"[Title/Abstract] OR "pan troglodytes"[Title/Abstract] OR "gibbon"[Title/Abstract] OR "gibbons"[Title/Abstract] OR "siamang"[Title/Abstract] OR "siamangs"[Title/Abstract] OR "nomascus"[Title/Abstract] OR "symphalangus"[Title/Abstract] OR "chimpanzee"[Title/Abstract] OR "chimpanzees"[Title/Abstract] OR "prosimian"[Title/Abstract] OR "prosimians"[Title/Abstract] OR "bush baby"[Title/Abstract] OR "bush babies"[Title/Abstract] OR "galagos"[Title/Abstract] OR "galago"[Title/Abstract] OR "pongidae"[Title/Abstract] OR "gorilla"[Title/Abstract] OR "gorillas"[Title/Abstract] OR "pongo pygmaeus"[Title/Abstract] OR "orangutan"[Title/Abstract] OR "orangutans"[Title/Abstract] OR "lemur"[Title/Abstract] OR "lemurs"[Title/Abstract] OR "lemuridae"[Title/Abstract] OR "horse"[Title/Abstract] OR "horses"[Title/Abstract] OR "equus"[Title/Abstract] OR "cow"[Title/Abstract] OR "calf"[Title/Abstract] OR "bull"[Title/Abstract] | 7,260,244 |
| #4 | #1 AND #2 AND #3 | 70 |

### Search strategy for Embase

| **Search** | **Query** | **Items found** |
| --- | --- | --- |
| #1  Melatonin | 'melatonin'/exp OR 'melatonin' OR 'melatonin receptor':ab,ti,kw OR 'tasimelteon':ab,ti,kw OR 'ramelteon':ab,ti,kw OR (s AND 20098):ab,ti,kw OR melatonin*:ab,ti,kw OR 's 20098':ab,ti,kw OR s20098:ab,ti,kw OR 'tik 301':ab,ti,kw OR tik301:ab,ti,kw OR circadin:ab,ti,kw OR 'ly 15635':ab,ti,kw OR rozerem:ab,ti,kw OR hetlioz:ab,ti,kw OR valdoxan:ab,ti,kw OR thymanax:ab,ti,kw OR melitor:ab,ti,kw OR melovine:ab,ti,kw OR '5 methoxy n acetyltryptamine':ab,ti,kw OR 'n acetyl 5 methoxytryptamine':ab,ti,kw OR 'vec 162':ab,ti,kw OR vec162:ab,ti,kw OR 'tak 375':ab,ti,kw OR tak375:ab,ti,kw OR mt:ab,ti,kw OR mlt:ab,ti,kw | 92,228 |
| #2  Osteoarthritis | 'osteoarthritis'/exp OR osteoarthriti*:ab,ti,kw OR 'osteo arthriti*':ab,ti,kw OR osteoarthrotic:ab,ti,kw OR osteoarthros*:ab,ti,kw OR coxarthrosis:ab,ti,kw OR oa:ab,ti,kw | 181,592 |
| #3  Animal | animal:de OR 'invertebrate'/exp OR 'amphibia'/exp OR 'fish'/exp OR 'boreoeutheria'/exp OR 'afrotheria'/exp OR 'dermoptera'/exp OR 'glires'/exp OR 'scandentia'/exp OR 'sauropsid'/exp OR 'laurasiatheria'/exp OR 'ungulate'/exp OR 'reptile'/exp OR 'cercopithecidae'/exp OR 'marsupial'/exp OR 'monotremate'/exp OR 'prosimian'/exp OR 'tarsiiform'/exp OR 'hylobatidae'/exp OR 'xenarthra'/exp OR 'platyrrhini'/exp OR 'chimpanzee'/exp OR 'gorilla'/exp OR 'orang utan'/exp OR 'homo neanderthalensis'/exp OR 'cephalochordata'/exp OR 'hyperotreti'/exp OR 'urochordata'/exp OR 'ambulacraria'/exp OR 'coelomata'/exp OR 'protostomia'/exp OR 'pseudocoelomata'/exp OR 'coelenterate'/exp OR 'mesozoa'/exp OR 'placozoa'/exp OR 'porifera'/exp OR 'juvenile animal'/exp OR 'male animal'/exp OR 'female animal'/exp OR 'primate'/de OR 'haplorhini'/de OR 'mammal'/de OR 'catarrhini'/de OR 'simian'/de OR 'ape'/de OR 'amniote'/de OR 'tetrapod'/de OR 'vertebrate'/de OR 'chordata'/de OR 'deuterostomia'/de OR 'bilateria'/de OR 'therian'/de OR 'hominid'/de OR 'euarchontoglires'/de OR 'placental mammals'/de | 7,520,298 |
| #4 | #1 AND #2 AND #3 | 122 |

## **Supplementary Table S2.** Explanations for the full-text article exclusions

| **SL/NO** | **Title** | **Reasons** |
| --- | --- | --- |
| 1 | Circadian production of melatonin in cartilage modifies rhythmic gene expression | In vitro |
| 2 | Changes in vascularity of cartilage endplate of degenerated intervertebral discs in response to melatonin administration in rats | Wrong population |
| 3 | Melatonin rescued interleukin 1β-impaired chondrogenesis of human mesenchymal stem cells | In vitro |
| 4 | Melatonin enhances chondrogenic differentiation of human mesenchymal stem cells | In vitro |
| 5 | Melatonin treatment combined with treadmill exercise accelerates muscular adaptation through early inhibition of CHOP-mediated autophagy in the gastrocnemius of rats with intra-articular collagenase-induced knee laxity | Wrong outcome✝ |
| 6 | Melatonin mediates protective effects on inflammatory response induced by interleukin-1 beta in human mesenchymal stem cells | In vitro |
| 7 | Melatonin enhances cartilage matrix synthesis by porcine articular chondrocytes | In vitro |
| 8 | Melatonin: Effects on Cartilage Homeostasis and Therapeutic Prospects in Cartilage-related Diseases | Review |

✝The outcomes that neither related to our primary outcomes nor secondary outcomes.

# **Sensitivity analyses**

## **Supplementary Table S3.** Leave-one-out sensitivity analyses on IL-1β

|  | **Pooled SMD [95% CI]** | **Between-study heterogeneity** |
| --- | --- | --- |
| \| **Overall** \| \| --- \| | -5.45[-6.78, -4.12] | P = 0.25; 28% |
| **Omitted study** | | |
| Jiang_2014 | -4.99[-6.59, -3.40] | P = 0.19; 42% |
| Huang_2010b | -5.12[-6.76, -3.48] | P = 0.13; 57% |
| Guo_2017 | -6.28[-7.94, -4.62] | P = -.81; 0% |

## **Supplementary Table S4.** Leave-one-out sensitivity analyses on TNF-α

|  | **Pooled MD [95% CI]** | **Between-study heterogeneity** |
| --- | --- | --- |
| \| **Overall** \| \| --- \| | -71.63[-127.20, -16.05] | P < 0.00001; 99% |
| **Omitted study** | | |
| Guo_2017 | -32.28[-91.19, 26.63] | P < 0.00001; 99% |
| Hong_2017 | -80.44[-235.49, 74.61] | P < 0.00001; 99% |
| Hong_2014 | -109.94[-206.05, -13.83] | P < 0.00001; 97% |

## **Supplementary Table S5.** Leave-one-out sensitivity analyses on MMP-13

|  | **Pooled SMD [95% CI]** | **Between-study heterogeneity** |
| --- | --- | --- |
| \| **Overall** \| \| --- \| | -5.08[-9.82, -0.34] | P < 0.0001; 90% |
| **Omitted study** | | |
| Guo_2017 | -5.52[-14.67, 3.62] | P < 0.0001; 94% |
| Hong_2017 | -2.79[-6.48, 0.89] | P = 0.01; 845 |
| Hong_2014 | -7.37[-12.80, -1.94] | P = 0.03; 79% |

## **Supplementary Table S6.** Leave-one-out sensitivity analyses on histological grading score

|  | **Pooled SMD [95% CI]** | **Between-study heterogeneity** |
| --- | --- | --- |
| \| **Overall** \| \| --- \| | -3.46[-5.24, -1.68] | p < 0.0001; 84% |
| **Omitted study** | | |
| Lim_2012 | -3.42[-5.12, -1.29] | P < 0.0001; 87% |
| Huang_2010b | -2.82[-4.47, -1.17] | P = 0.002; 80% |
| Huang_2010a | -3.07[-4.93, -1.20] | P = 0.0005; 83% |
| Jiang_2014 | -4.11[-6.69, -1.52] | P < 0.0001; 87% |
| Zhang_2019 | -4.18[-6.52, -1.84] | P = 0.0005; 83% |

## **Supplementary Table S7.** Leave-one-out sensitivity analyses on serum melatonin level at 2 AM

|  | **Pooled MD [95% CI]** | **Between-study heterogeneity** |
| --- | --- | --- |
| \| **Overall** \| \| --- \| | -150.63[-168.64, -132.62] | P = 0.08; 61% |
| **Omitted study** | | |
| Huang_2010a | -145.62[-169.43, -121.81] | P = 0.06; 71% |
| Huang_2010c | -159.67[-173.73, -145.62] | P = 0.74; 0% |
| Jiang_2014 | -147.24[-175.80, -118.68] | P = 0.05; 75% |

## **Supplementary Table S8.** Leave-one-out sensitivity analyses on serum melatonin level at 2 PM

|  | **Pooled MD [95% CI]** | **Between-study heterogeneity** |
| --- | --- | --- |
| \| **Overall** \| \| --- \| | -16.91[-23.57, -10.25] | P = 0.11; 55% |
| **Omitted study** | | |
| Huang_2010a | -14.81[-23.08, -6.54] | P = 0.11; 61% |
| Huang_2010c | -20.20[-25.78, -14.62] | P = 0.62; 0% |
| Jiang_2014 | -15.97[-27.02, -4.92] | P = 0.05; 74% |
